# Supplementary material for: Addressing the mean–variance relationship in spatially resolved transcriptomics data with spoon
Source: Biostatistics. 2025 Jun 14;26(1):kxaf012. doi: 10.1093/biostatistics/kxaf012 (PMC12166475; doi:10.1093/biostatistics/kxaf012)
Supplement: kxaf012_Supplementary_Data [file kxaf012_supplementary_data.zip › biosts-24390-File002.pdf]

Supplementary Materials

Addressing the mean-variance relationship in spatially resolved transcriptomics data with *spoon*

Kinnary Shah, Boyi Guo, Stephanie C. Hicks*

*Correspondence to `shicks19@jhu.edu`

The Supplementary Materials include eight figures and eight tables referenced throughout the Main Manuscript. The figures expand on the results presented in the Main Manuscript, further illustrating the impacts of the mean-variance relationship and providing additional context for our simulations. The tables supplement Figure 5 by listing citations for all of the genes associated with cancer in each subcategory of the four cancer datasets.

Contents

1. **Figures S1-S8**
2. **Tables S1-S8**

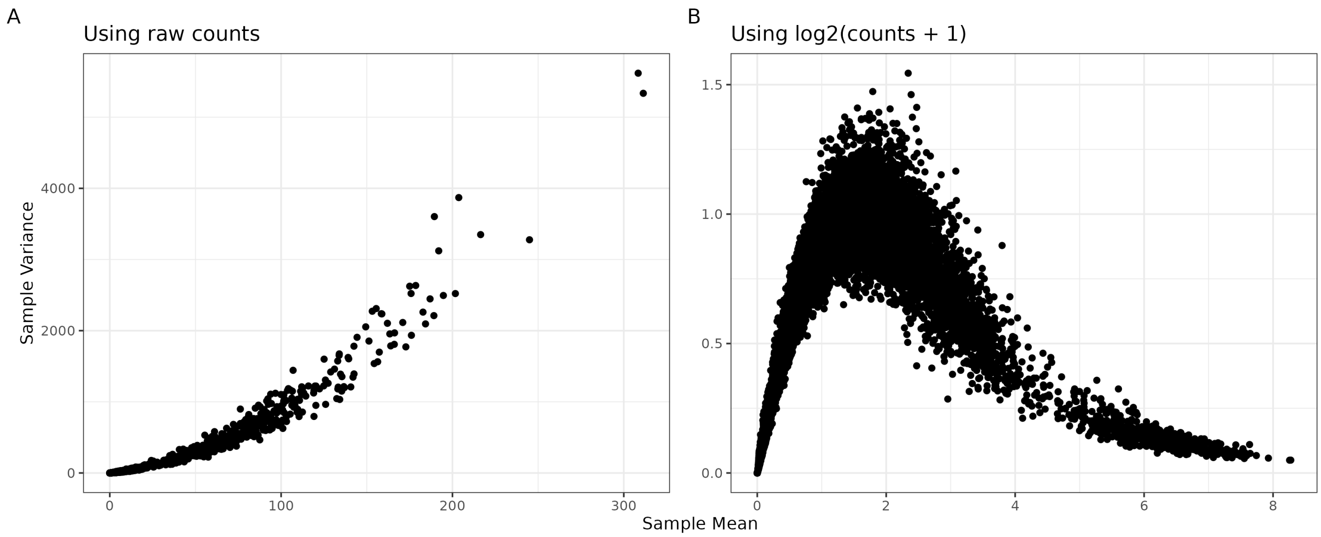

Figure S1: Visualizing the mean-variance relationship on different scales. The mean-variance relationship exists with or without a log-transformation. Gene expression counts were simulated using the `splatter` R/Bioconductor package [1] for G=10,000 genes and N=100 observations (or cells) under a Gamma-Poisson model. Each point represents one gene. Both representations illustrate the mean-variance relationship where the x-axis is the sample mean and y-axis is the sample variance using either (**A**) the raw counts or (**B**) the $\log_2$-transformed counts with a pseudocount of 1 (or $\log_2(\text{counts}+1)$). Here, the log-transformation overcorrects for the mean-variance relationship for the larger counts.

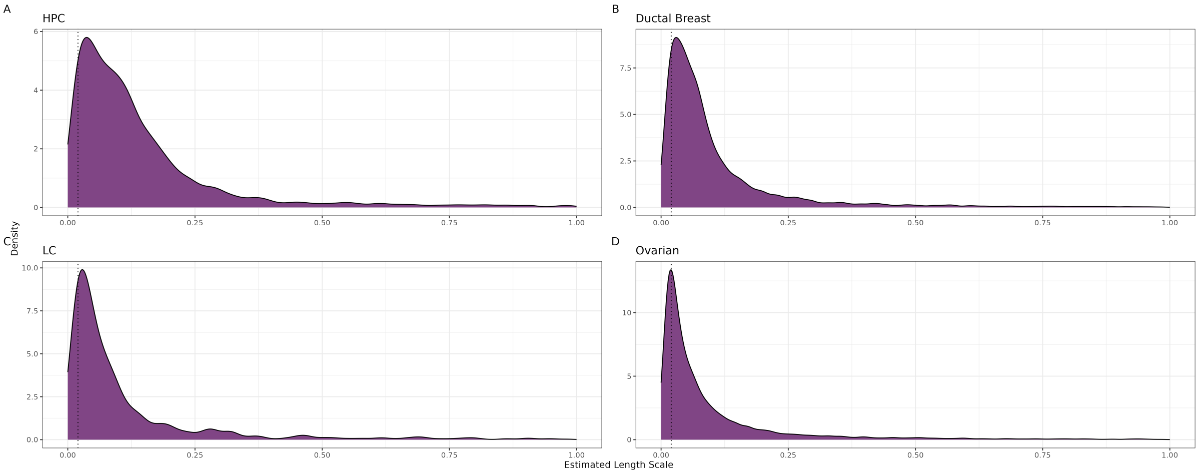

Figure S2: Real data estimated lengthscale distributions using `nnSVG`. This figure shows the estimated lengthscale distributions for four real datasets (**A**) HPC [2], (**B**) Ductal Breast cancer [3], (**C**) LC [4], and (**D**) Ovarian cancer [5]. For each dataset, `nnSVG` was used to calculate the estimated lengthscale value for each gene and the distribution of values between 0 and 1 is plotted. The dotted line highlights the lengthscale value used in the primary simulation evaluations.

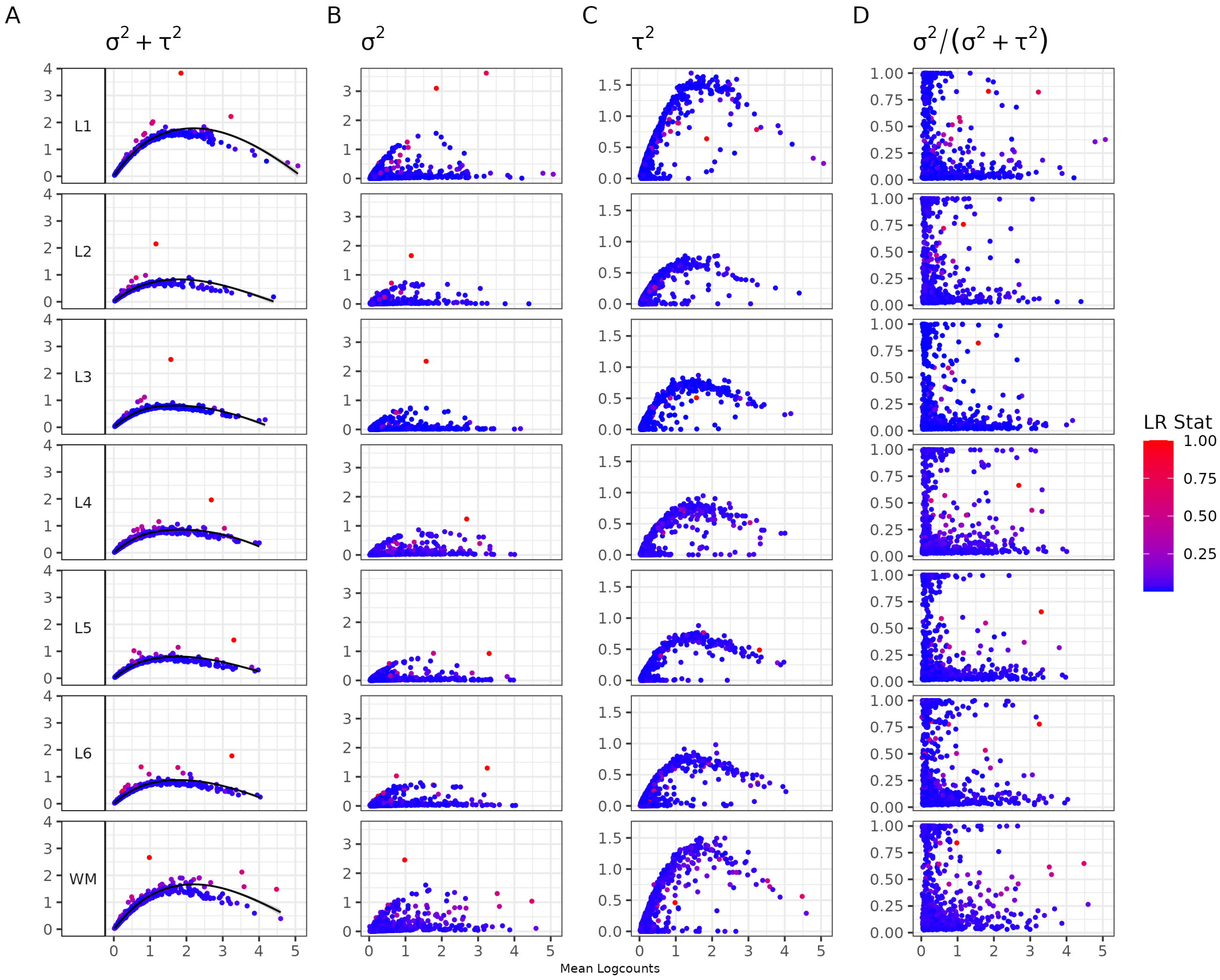

Figure S3: Mean-variance relationship after conditioning out biological variance measured by Gaussian process. Each row is a cortical layer from the DLPFC dataset, in order from top to bottom: Layers I-VI, white matter (WM). Each point is a gene colored by the likelihood ratio statistic (LR Stat) for a test comparing the fitted model against a classical linear model for the spatial component of variance. The likelihood ratio statistics are scaled by the maximum likelihood ratio statistic for each layer in order to have more uniform visualization. The x-axis represents mean logcounts and the y-axes represent different components of variance, in order from left to right: total variance $\sigma^2 + \tau^2$, spatial variance σ^2, nonspatial variance τ^2, and proportion of spatial variance $\sigma^2/(\sigma^2 + \tau^2)$.

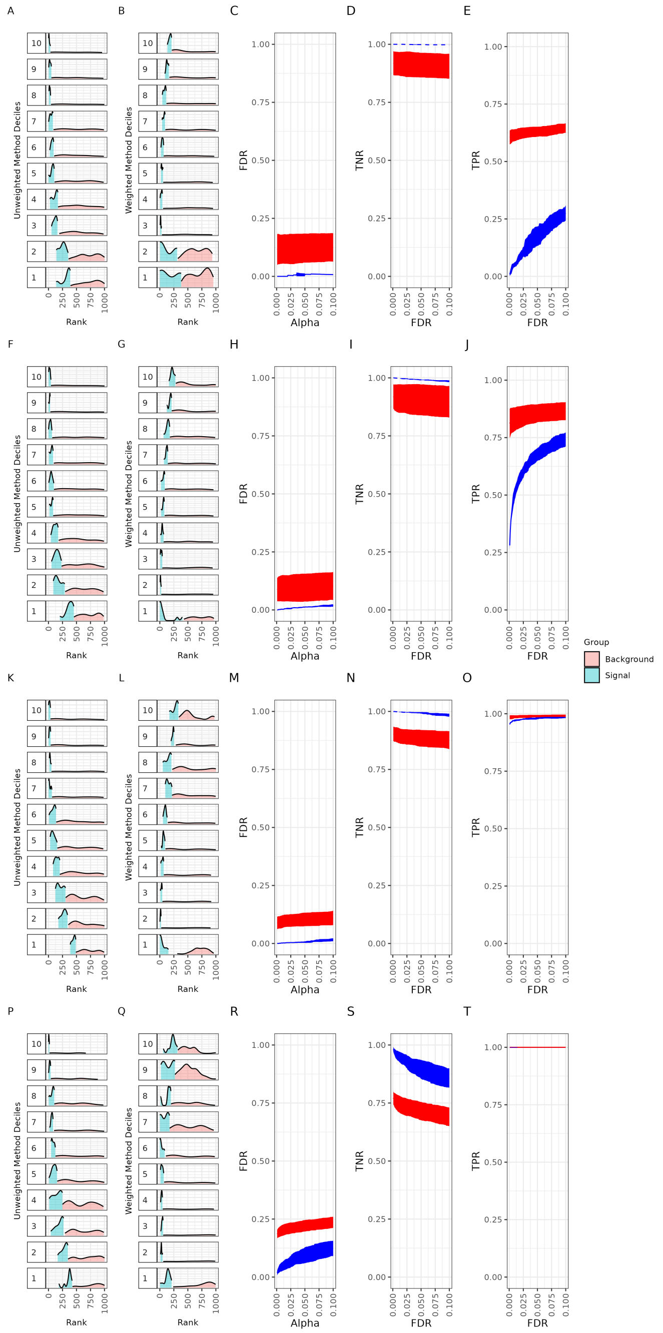

Figure S4: Removing the mean-variance relationship with expanded lengthscale metrics. This dataset contains 1,000 simulated genes across 968 spots. Each row represents a simulation setting with unique lengthscales, in order from top to bottom: 50, 60, 100, 500. Separately for unweighted and weighted methods, the genes were binned into deciles based on mean logcounts. Decile 1 is the lowest mean expression values. The first column of plots is unweighted ranks and the second column of plots is weighted ranks. Within each decile, the density of the top 10% ranks is plotted as the signal and the density of the remaining ranks is plotted as the background. The final three columns show the false discovery rate (FDR), true negative rate (TNR), and true positive rate (TPR). The red represents weighted nnSVG and the blue represents unweighted nnSVG. These plots represent the average of each respective rate over five iterations of the same simulation with unique random seeds.

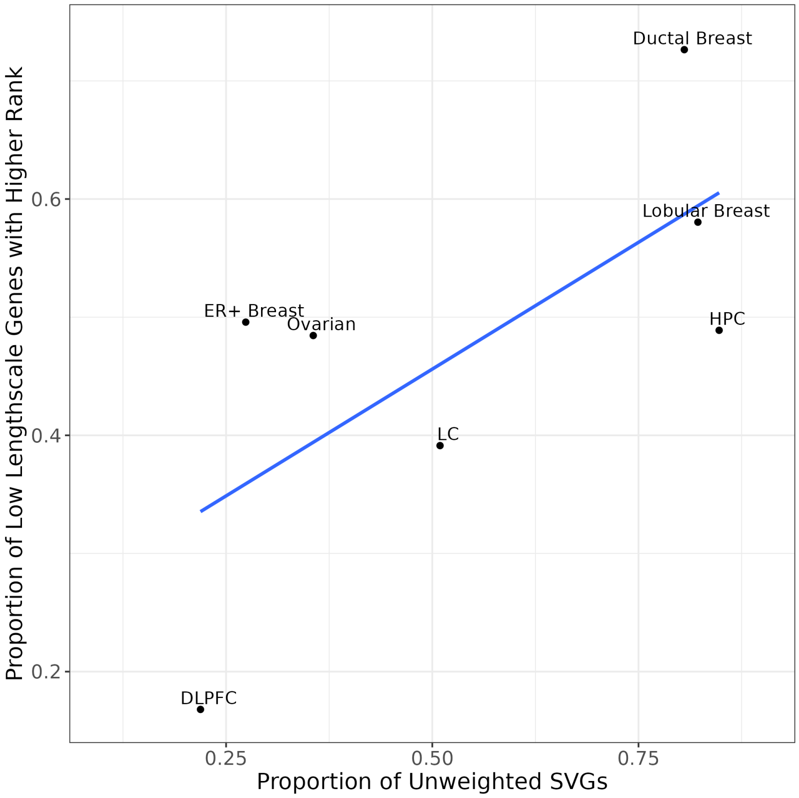

Figure S5: Ranking small lengthscale genes after weighting. Each point is a unique real dataset analyzed with 10x Genomics Visium. The x-axis is the proportion of SVGs from running unweighted `nnSVG` on the dataset. The y-axis is the proportion of genes with a small lengthscale (40-90) that are higher ranked in weighted `nnSVG` compared to unweighted `nnSVG`.

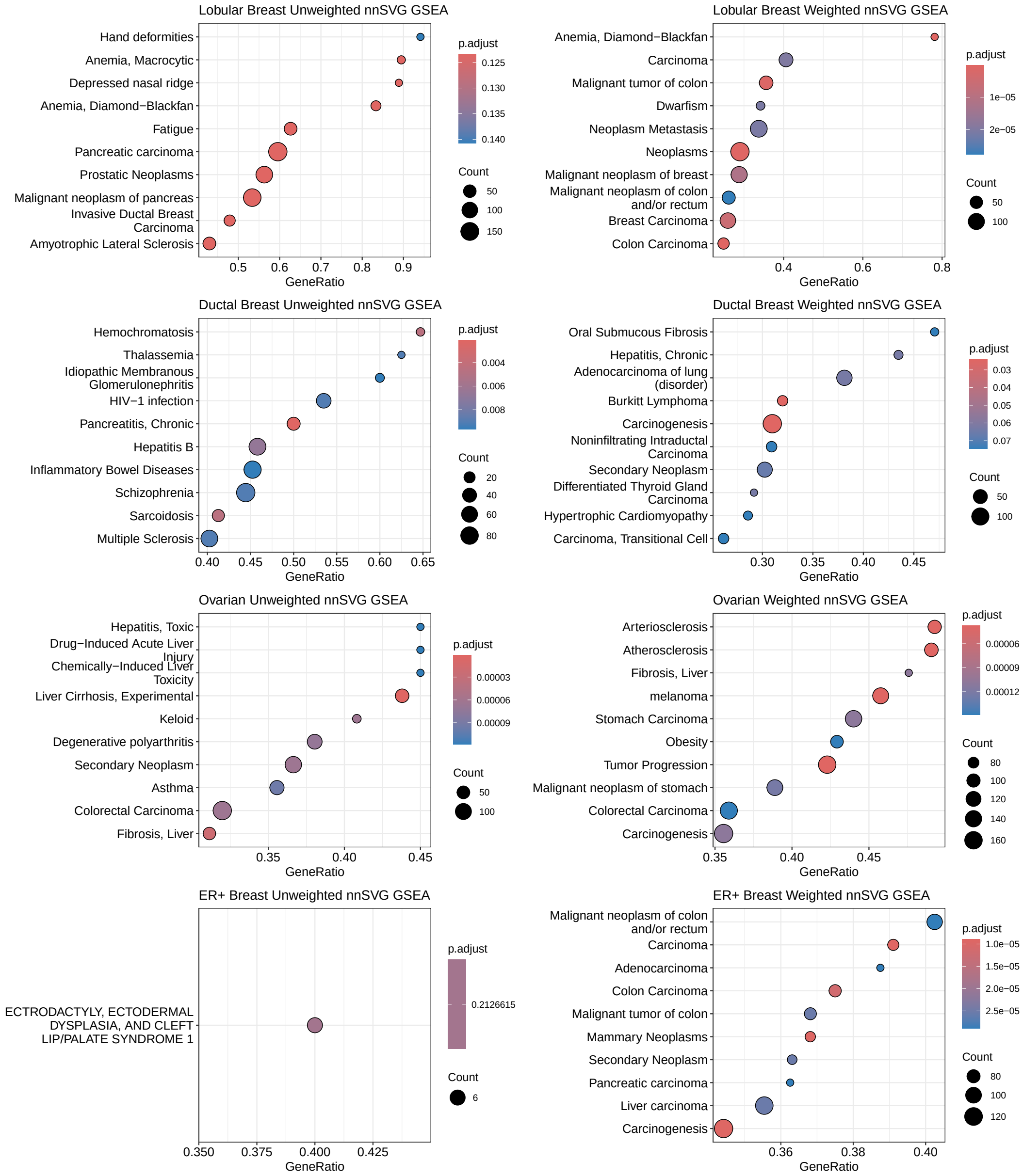

Figure S6: Gene set enrichment analysis (GSEA) using Disease Gene Network (DisGeNet) on cancer datasets before and after weighting. Each row shows two GSEA analyses for each of the four cancer datasets. The first column uses the unweighted SVG ranks of all genes, while the second column uses the weighted SVG ranks for all genes.

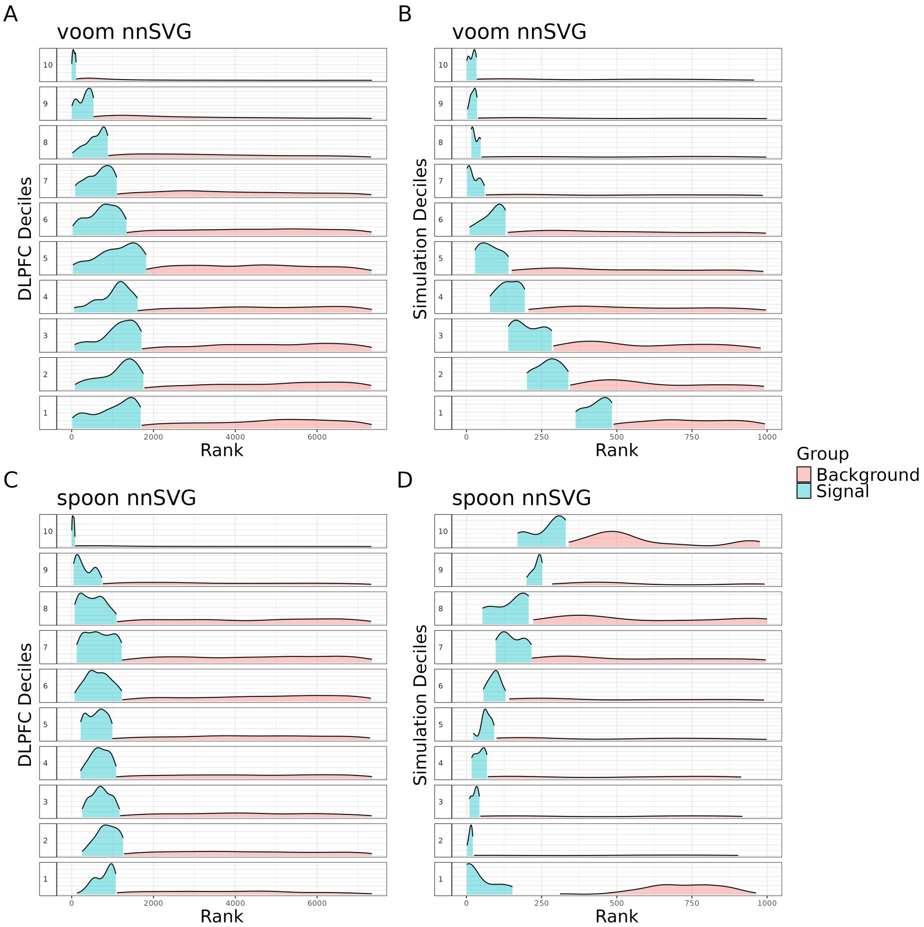

Figure S7: Comparison of limma-voom to *spoon* weights with nnSVG. The first column shows the DLPFC dataset and the second column shows a simulated dataset following the framework outlined in the main text. The first row shows nnSVG ranks for the two datasets with limma-voom weights, and the second row shows nnSVG ranks for the two datasets with *spoon* ranks.

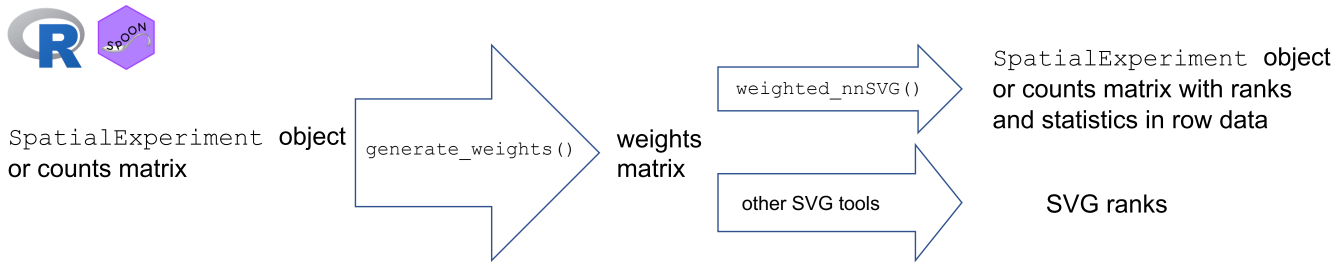

Figure S8: Schematic of *spoon* software. This schematic describes inputs and outputs of the *spoon* software.

Table S1: Low Mean, Ductal Breast Genes Relation to Cancer. This table shows all genes with means less than the 25th percentile in the Ductal Breast [3] dataset which were in the lowest 10% of ranks before weighting and then increased to the highest 10% of ranks after weighting. The second column indicates if the gene is related to Breast cancer, with the corresponding reference in the third column.

Gene	Cancer-related?	References
TMEM39B		
ETAA1		
ATXN7	TRUE	[6]
BBS7		
MFSD8		
ETFDH		
E2F3	TRUE	[7]
FIG4		
TSPYL4		
METTL2B		
MFHAS1	TRUE	[8]
MAK16		
GKAP1		
SAAL1	TRUE	[9]
DDX10	TRUE	[10]
B3GLCT		
GNB5		
TLN2	TRUE	[11]
VPS33B		
ASB7		
MOSMO		
MED26		
ZNF227		
ITCH	TRUE	[12]
ZHX3	TRUE	[13]
ZBTB21		

Table S2: Low Mean, ER+ Breast Genes Relation to Cancer. This table shows all genes with means less than the 25th percentile in the ER+ Breast [14] dataset which were in the lowest 10% of ranks before weighting and then increased to the highest 10% of ranks after weighting. The second column indicates if the gene is related to Breast cancer, with the corresponding reference in the third column.

Gene	Cancer-related?	References
RELA-DT		

Table S3: Low Mean, Lobular Breast Genes Relation to Cancer. This table shows all genes with means less than the 25th percentile in the Lobular Breast [15] dataset which were in the lowest 10% of ranks before weighting and then increased to the highest 10% of ranks after weighting. The second column indicates if the gene is related to Breast cancer, with the corresponding reference in the third column.

Gene	Cancer-related?	References
TMEM51		
PDK1	TRUE	[16]
QTRT2		
OSBPL11		
TMEM44		
CPLX1		
PPAT	TRUE	[17]
ATP6AP1L	TRUE	[18]
LYRM7	TRUE	[19]
MSH5		
ZBTB24		
SHPRH		
TRIM35	TRUE	[20]
NCALD		
SNX30		
TRIM32	TRUE	[21]
FRAT1	TRUE	
RAB11FIP2		
TIGAR	TRUE	[22]
INTS13		
DLEU2		
ATXN1L		
SNHG30		
SEPTIN4		
ZNF439	TRUE	[23]
ZNF181		
TMEM191B		
ZNF74		
MOSPD2	TRUE	[24]
FAAH2		
BRWD3	TRUE	[25]

Table S4: Low Mean, Ovarian Genes Relation to Cancer. This table shows all genes with means less than the 25th percentile in the Ovarian [5] dataset which were in the lowest 10% of ranks before weighting and then increased to the highest 10% of ranks after weighting. The second column indicates if the gene is related to Ovarian cancer, with the corresponding reference in the third column.

Gene	Cancer-related?	References
TUFT1	TRUE	[26]
EHHADH		
DDX39B	TRUE	[27]
NAV2		
AC026471.4		
SMYD4		
HEXIM2		

Table S5: Small Lengthscale, Ductal Breast Genes Relation to Cancer. This table shows all genes with lengthscale values between 40 to 90 in the Ductal Breast [3] dataset which were ranked higher after weighting. The second column indicates if the gene is related to Breast cancer, with the corresponding reference in the third column.

Gene	Cancer-related?	References
S100PBP		
MED8		
DDX20	TRUE	[28]
GPR89A		
ASXL2	TRUE	[29]
PARTICL		
IMP4		
MGAT5	TRUE	[30]
ICOS	TRUE	[31]
AC112220.2		
PHF7		
ATXN7		
DNAJC13		
GAPT		
AC008608.2		
AC106795.2		
SAPCD1		
TNFRSF21	TRUE	[32]
CRYBG1		
MOXD1		
NUP43	TRUE	[33]
CDK13	TRUE	[34]
ZKSCAN1		
CLDN15		
NOM1		
NOL6	TRUE	[35]
ZCCHC7		
DAPK1	TRUE	[36]
MFSD14B		
CIZ1		
BORCS7		
TRIM21	TRUE	[37]
APIP		
FAM111B	TRUE	[38]
SPDYC	TRUE	[39]
AP5B1		
SPTBN2		
P2RY6		
CWC15		
CLEC4A		
AC087239.1		
ETFBKMT		
ZC3H10		
CRYL1		
IFT88		
MIS18BP1		
ZBTB1	TRUE	[40]
ERG28		
PEAK1	TRUE	[41]
RCCD1	TRUE	[42]
TEDC2		
NDE1		
FTO	TRUE	[43]
B3GNT9		
PARD6A		
SNAI3-AS1		
ALOX15		
ULK2	TRUE	[44]
TANC2		
TRIM65	TRUE	[45]
UBE2O		
ZNF532		
ZNF557		
MAP2K7		
ZNF490		
ZNF429		
C19orf47		
IRF2BP1		
AC010331.1		
DNMT3B	TRUE	[46]
PPP1R3D		
SEC14L2		
PMM1		
CRELD2		
ZMAT1		
NKRF		
MMGT1		

Table S6: Small Lengthscale, ER+ Breast Genes Relation to Cancer. This table shows all genes with lengthscale values between 40 to 90 in the ER+ Breast [14] dataset which were ranked higher after weighting. The second column indicates if the gene is related to Breast cancer, with the corresponding reference in the third column.

Gene	Cancer-related?	References
MTFR1L		
PRPF38A		
TBCE		
KIAA1841		
TSN		
CASP8	TRUE	[47]
AC022007.1		
HEMK1		
TRMT10C		
PLD1		
ATP11B	TRUE	[48]
YEATS2		
E2F3	TRUE	[7]
EHMT2	TRUE	[49]
PRRT1		
TREM1	TRUE	[50]
MSC		
SMC2	TRUE	[51]
CIZ1		
GTF3C4		
BRD3OS		
SEC31B		
CD82	TRUE	[52]
BIRC2	TRUE	[53]
UPK2		
HMBS		
GABARAPL1	TRUE	[54]
STAT2		
NUAK1		
RHOF		
SLC7A1		
EFCAB11		
ZNF770		
HOMER2		
POLR2C		
CDYL2	TRUE	[55]
MBTPS1		
CTU2		
DRG2		
BLMH		
RASL10B		
NBR2		
MSI2	TRUE	[56]
QRICH2		
ME2		
CTDP1	TRUE	[57]
DDA1	TRUE	[58]
SLC25A42		
AC006504.5		
STRN4		
TTYH1		
ZNF544		
JAM2	TRUE	[59]
AC245060.5		
NOL12		
GTPBP1		
TAF1	TRUE	[60]
FGF13	TRUE	[61]
LINC00893		

Table S7: Small Lengthscale, Lobular Breast Genes Relation to Cancer. This table shows all genes with lengthscale values between 40 to 90 in the Lobular Breast [15] dataset which were ranked higher after weighting. The second column indicates if the gene is related to Breast cancer, with the corresponding reference in the third column.

Gene	Cancer-related?	References
FBXO2		
IFFO2		
ZNF436	TRUE	[62]
EXTL1		
MTF2	TRUE	[63]
PHTF1		
HAGLR	TRUE	[64]
ORC2		
SATB1	TRUE	[65]
C3orf38		
CMSS1		
CD200	TRUE	[66]
TRIM59	TRUE	[67]
ETV5		
UCHL1	TRUE	[68]
INTS12		
LIFR	TRUE	[69]
ZBED3		
FER	TRUE	[70]
DCP2		
MIR3936HG		
SH3RF2		
SLC36A1		
BNIP1		
HIST1H4J		
NHSL1		
C7orf25		
HUS1		
ZSCAN21		
AASS		
AC004918.1		
FANCG		
NFIL3	TRUE	[71]
AKNA		
PROSER2		
THNSL1		
ENTPD1	TRUE	[72]
CWF19L1		
ARMH3		
ROM1		
AP000438.1		
FDXACB1		
CLEC2D	TRUE	[73]
RASSF3		
PRDM4		
FLT1	TRUE	[74]
BIVM		
ZNF219		
NYNRIN		
CGRRF1	TRUE	[75]
SGPP1		
FAM71D		
GABRB3		
CHST14		
TYRO3	TRUE	[76]
ADAM10	TRUE	[77]
GRAMD2A		
ASPHD1		
PLEKHG4		
PDPR		
MLYCD		
ZC3H18		
CENPBD1		
NLRP1	TRUE	[78]
PIGW		
CEP95		
ABCA5		
MEX3C		
ZNF77		
ZGLP1		
HAUS5	TRUE	[79]
ZNF574		
ZNF628		
ZNF579		
ELMO2		
DIP2A		
PLA2G3		
KCTD17		
APOBEC3C		
TCF20		
JADE3		
TRO		
MECP2	TRUE	[80]

Table S8: Small Lengthscale, Ovarian Genes Relation to Cancer. This table shows all genes with lengthscale values between 40 to 90 in the Ovarian [5] dataset which were ranked higher after weighting. The second column indicates if the gene is related to Ovarian cancer, with the corresponding reference in the third column.

Gene	Cancer-related?	References
HYI		
ECHDC2		
RAVER2		
WDR3		
PAQR6		
DESI2		
STRN		
ACYP2		
PAIP2B		
LIMD1		
PRICKLE2		
BBX		
LSAMP	TRUE	[81]
TMEM39A		
DTX3L		
B3GNT5		
IQCG		
LIN54		
PDE5A		
LARP1B		
HPF1		
DCP2		
WDR55		
CASC15	TRUE	[82]
SLC18B1		
ICA1		
PSPH		
RCC1L		
COG5		
WASL		
KLHDC10		
ZNF775		
INSIG1		
SLC25A6	TRUE	[83]
CXorf36		
HNRNPH2		
PRPS1		
SGK3		
AF117829.1		
ZC3H3		
NPR2		
SCAI		
ZBTB34		
CDK9	TRUE	[84]
LRRC56		
INTS5		
HIKESHI		
CASP1		
SIDT2		
MAP3K8	TRUE	[85]
ERCC6		
FAM149B1		
NOC3L		
LRRC27		
LRRC23		
RDH5		
ARHGEF25		
TXNRD1		
MVK		
B3GLCT		
GCH1	TRUE	[86]
C16orf87		
ZNF319		
SLC12A4		
AC008105.3		
PCYT2	TRUE	[87]
TRAPPC8		
SDCBP2		
BSG		
ZBTB7A		
ZNF443		
ASF1B		
ZNF135		
ATP6V1E1		
ZNRF3		
NOL12		
SREBF2		

References

[1] L. Zappia, B. Phipson, and A. Oshlack. Splatter: simulation of single-cell RNA sequencing data. 18 (1):174. ISSN 1474-760X. doi:10.1186/s13059-017-1305-0. URL http://genomebiology.biomedcentral.com/articles/10.1186/s13059-017-1305-0.

[2] E. D. Nelson, M. Tippani, A. D. Ramnauth, H. R. Divecha, R. A. Miller, N. J. Eagles, E. A. Pattie, S. H. Kwon, S. V. Bach, U. M. Kaipa, J. Yao, J. E. Kleinman, L. Collado-Torres, S. Han, K. R. Maynard, T. M. Hyde, K. Martinowich, S. C. Page, and S. C. Hicks. An integrated single-nucleus and spatial transcriptomics atlas reveals the molecular landscape of the human hippocampus. URL http://biorxiv.org/lookup/doi/10.1101/2024.04.26.590643.

[3] 10x Genomics. Human breast cancer: Visium fresh frozen, whole transcriptome, . URL https://www.10xgenomics.com/resources/datasets/human-breast-cancer-visium-fresh-frozen-whole-transcriptome-1-standard.

[4] L. M. Weber, H. R. Divecha, M. N. Tran, S. H. Kwon, A. Spangler, K. D. Montgomery, M. Tippani, R. Bharadwaj, J. E. Kleinman, S. C. Page, T. M. Hyde, L. Collado-Torres, K. R. Maynard, K. Martinowich, and S. C. Hicks. The gene expression landscape of the human locus coeruleus revealed by single-nucleus and spatially-resolved transcriptomics. 12. doi:10.7554/eLife.84628. URL https://elifesciences.org/reviewed-preprints/84628.

[5] E. Denisenko, L. de Kock, A. Tan, A. B. Beasley, M. Beilin, M. E. Jones, R. Hou, D. O. Muiri, S. Bilic, G. R. K. A. Mohan, S. Salfinger, S. Fox, K. P. W. Hmon, Y. Yeow, Y. Kim, R. John, T. S. Gilderman, E. Killingbeck, E. S. Gray, P. A. Cohen, Y. Yu, and A. R. R. Forrest. Spatial transcriptomics reveals discrete tumour microenvironments and autocrine loops within ovarian cancer subclones. 15(1):2860. ISSN 2041-1723. doi:10.1038/s41467-024-47271-y. URL https://www.nature.com/articles/s41467-024-47271-y.

[6] R. L. Milne, B. Burwinkel, K. Michailidou, J.-I. Arias-Perez, M. P. Zamora, P. Menéndez-Rodríguez, D. Hardisson, M. Mendiola, A. González-Neira, G. Pita, M. R. Alonso, J. Dennis, Q. Wang, M. K. Bolla, A. Swerdlow, A. Ashworth, N. Orr, M. Schoemaker, Y.-D. Ko, H. Brauch, U. Hamann, I. L. Andrulis, J. A. Knight, G. Glendon, S. Tchatchou, K. Matsuo, H. Ito, H. Iwata, K. Tajima, J. Li, J. S. Brand, H. Brenner, A. K. Dieffenbach, V. Arndt, C. Stegmaier, D. Lambrechts, G. Peuteman, M.-R. Christiaens, A. Smeets, A. Jakubowska, J. Lubinski, K. Jaworska-Bieniek, K. Durda, M. Hartman, M. Hui, W. Yen Lim, C. Wan Chan, F. Marme, R. Yang, P. Bugert, A. Lindblom, S. Margolin, M. García-Closas, S. J. Chanock, J. Lissowska, J. D. Figueroa, S. E. Bojesen, B. G. Nordestgaard, H. Flyger, M. J. Hooning, M. Kriege, A. M. van den Ouweland, L. B. Koppert, O. Fletcher, N. Johnson, I. dos Santos-Silva, J. Peto, W. Zheng, S. Deming-Halverson, M. J. Shrubsole, J. Long, J. Chang-Claude, A. Rudolph, P. Seibold, D. Flesch-Janys, R. Winqvist, K. Pylkäs, A. Jukkola-Vuorinen, M. Grip, A. Cox, S. S. Cross, M. W. Reed, M. K. Schmidt, A. Broeks, S. Cornelissen, L. Braaf, D. Kang, J.-Y. Choi, S. K. Park, D.-Y. Noh, J. Simard, M. Dumont, M. S. Goldberg, F. Labrèche, P. A. Fasching, A. Hein, A. B. Ekici, M. W. Beckmann, P. Radice, P. Peterlongo, J. Azzollini, M. Barile, E. Sawyer, I. Tomlinson, M. Kerin, N. Miller, J. L. Hopper, D. F. Schmidt, E. Makalic, M. C. Southey, S. Hwang Teo, C. Har Yip, K. Sivanandan, W.-T. Tay, C.-Y. Shen, C.-N. Hsiung, J.-C. Yu, M.-F. Hou, P. Guénel, T. Truong, M. Sanchez, C. Mulot, W. Blot, Q. Cai, H. Nevanlinna, T. A. Muranen, K. Aittomäki, C. Blomqvist, A. H. Wu, C.-C. Tseng, D. Van Den Berg, D. O. Stram, N. Bogdanova, T. Dörk, K. Muir, A. Lophatananon,

S. Stewart-Brown, P. Siriwanarangsan, A. Mannermaa, V. Kataja, V.-M. Kosma, J. M. Hartikainen, X.-O. Shu, W. Lu, Y.-T. Gao, B. Zhang, F. J. Couch, A. E. Toland, D. Yannoukakos, S. Sangrajrang, J. McKay, X. Wang, J. E. Olson, C. Vachon, K. Purrington, G. Severi, L. Baglietto, C. A. Haiman, B. E. Henderson, F. Schumacher, L. Le Marchand, P. Devilee, R. A. Tollenaar, C. Seynaeve, K. Czene, M. Eriksson, K. Humphreys, H. Darabi, S. Ahmed, M. Shah, P. D. Pharoah, P. Hall, G. G. Giles, J. Benítez, A. M. Dunning, G. Chenevix-Trench, D. F. Easton, A. Berchuck, R. A. Eeles, A. A. A. Olama, Z. Kote-Jarai, S. Benlloch, A. Antoniou, L. McGuffog, K. Offit, A. Lee, E. Dicks, C. Luccarini, D. C. Tessier, F. Bacot, D. Vincent, S. LaBoissière, F. Robidoux, S. F. Nielsen, J. M. Cunningham, S. A. Windebank, C. A. Hilker, J. Meyer, M. Angelakos, J. Maskiell, E. van der Schoot, E. Rutgers, S. Verhoef, F. Hogervorst, P. Boonyawongviroj, P. Siriwanarungsan, M. Schrauder, M. Rübner, S. Oeser, S. Landrith, E. Williams, E. Ryder-Mills, K. Sargus, N. McInerney, G. Colleran, A. Rowan, A. Jones, C. Sohn, A. Schneeweiß, P. Bugert, N. Álvarez, J. Lacey, S. Wang, H. Ma, Y. Lu, D. Deapen, R. Pinder, E. Lee, F. Schumacher, P. Horn-Ross, P. Reynolds, D. Nelson, H. Ziegler, S. Wolf, V. Hermann, W.-Y. Lo, C. Justenhoven, C. Baisch, H.-P. Fischer, T. Brüning, B. Pesch, S. Rabstein, A. Lotz, V. Harth, T. Heikkinen, I. Erkkilä, K. Aaltonen, K. von Smitten, N. Antonenkova, P. Hillemanns, H. Christiansen, E. Myöhänen, H. Kemiläinen, H. Thorne, E. Niedermayr, D. Bowtell, G. Chenevix-Trench, A. deFazio, D. Gertig, A. Green, P. Webb, A. Green, P. Parsons, N. Hayward, P. Webb, D. Whiteman, A. Fung, J. Yashiki, G. Peuteman, D. Smeets, T. V. Brussel, K. Corthouts, N. Obi, J. Heinz, S. Behrens, U. Eilber, M. Celik, T. Olchers, S. Manoukian, B. Peissel, G. Scuvera, D. Zaffaroni, B. Bonanni, I. Feroce, A. Maniscalco, A. Rossi, L. Bernard, M. Tranchant, M.-F. Valois, A. Turgeon, L. Heguy, P. Sze Yee, P. Kang, K. I. Nee, S. Mariapun, Y. Sook-Yee, D. Lee, T. Y. Ching, N. A. M. Taib, M. Otsukka, K. Mononen, T. Selander, N. Weerasooriya, O. staff, E. Krol-Warmerdam, J. Molenaar, J. Blom, L. Brinton, N. Szeszenia-Dabrowska, B. Peplonska, W. Zatonski, P. Chao, M. Stagner, P. Bos, J. Blom, E. Crepin, A. Nieuwlaat, A. Heemskerk, S. Higham, S. Cross, H. Cramp, D. Connley, S. Balasubramanian, I. Brock, C. Luccarini, D. Conroy, C. Baynes, and K. Chua. Common non-synonymous SNPs associated with breast cancer susceptibility: findings from the breast cancer association consortium. 23(22):6096–6111. ISSN 0964-6906. doi:10.1093/hmg/ddu311. URL `https://www.ncbi.nlm.nih.gov/pmc/articles/PMC4204770/`.

[7] S. Jusino, Y. Rivera-Rivera, C. Chardón-Colón, A. J. Ruiz-Justiz, J. Vélez-Velázquez, A. Isidro, M. E. Cruz-Robles, M. Bonilla-Claudio, G. N. Armaiz-Pena, and H. I. Saavedra. E2f3 drives the epithelial-to-mesenchymal transition, cell invasion, and metastasis in breast cancer. 246(19): 2057–2071. ISSN 1535-3702, 1535-3699. doi:10.1177/15353702211035693. URL `http://journals.sagepub.com/doi/10.1177/15353702211035693`.

[8] Y. Jin, M. Zhai, R. Cao, H. Yu, C. Wu, and Y. Liu. Silencing MFHAS1 induces pyroptosis via the JNK-activated NF-KB/caspase1/ GSDMD signal axis in breast cancer. 29(42):3408–3420, . ISSN 1873-4286. doi:10.2174/0113816128268130231026054649.

[9] W. Yang, B. Han, Y. Chen, and F. Geng. SAAL1, a novel oncogene, is associated with prognosis and immunotherapy in multiple types of cancer. 14(15):6316, . doi:10.18632/aging.204224. URL `https://www.ncbi.nlm.nih.gov/pmc/articles/PMC9417231/`.

[10] X. Jiao, S. D. Hooper, T. Djureinovic, C. Larsson, F. Wärnberg, C. Tellgren-Roth, J. Botling, and T. Sjöblom. Gene rearrangements in hormone receptor negative breast cancers revealed by mate pair sequencing. 14:165. ISSN 1471-2164. doi:10.1186/1471-2164-14-165. URL `https://www.ncbi.nlm.nih.gov/pmc/articles/PMC3600027/`.

[11] L. Li, X. Li, L. Qi, P. Rychahou, N. Jafari, and C. Huang. The role of talin2 in breast cancer tumorigenesis and metastasis. 8(63):106876, . doi:10.18632/oncotarget.22449. URL https://www.ncbi.nlm.nih.gov/pmc/articles/PMC5739781/.

[12] Q. Yin, C. J. Wyatt, T. Han, K. S. Smalley, and L. Wan. ITCH as a potential therapeutic target in human cancers. 67:117–130. ISSN 1044-579X. doi:10.1016/j.semcancer.2020.03.003. URL https://www.ncbi.nlm.nih.gov/pmc/articles/PMC7724637/.

[13] Y. You, Y. Ma, Q. Wang, Z. Ye, Y. Deng, and F. Bai. Attenuated ZHX3 expression serves as a potential biomarker that predicts poor clinical outcomes in breast cancer patients. 11:1199–1210. ISSN 1179-1322. doi:10.2147/CMAR.S184340. URL https://www.ncbi.nlm.nih.gov/pmc/articles/PMC6368119/.

[14] S. Z. Wu, G. Al-Eryani, D. Roden, S. Junankar, K. Harvey, A. Andersson, A. Thennavan, C. Wang, J. Torpy, N. Bartonicek, T. Wang, L. Larsson, D. Kaczorowski, N. I. Weisenfeld, C. R. Uytingco, J. G. Chew, Z. W. Bent, C.-L. Chan, V. Gnanasambandapillai, C.-A. Dutertre, L. Gluch, M. N. Hui, J. Beith, A. Parker, E. Robbins, D. Segara, C. Cooper, C. Mak, B. Chan, S. Warrier, F. Ginhoux, E. Millar, J. E. Powell, S. R. Williams, X. S. Liu, S. O'Toole, E. Lim, J. Lundeberg, C. M. Perou, and A. Swarbrick. A single-cell and spatially resolved atlas of human breast cancers. 53(9): 1334–1347, . ISSN 1061-4036. doi:10.1038/s41588-021-00911-1. URL https://www.ncbi.nlm.nih.gov/pmc/articles/PMC9044823/.

[15] 10x Genomics. Human breast cancer: Whole transcriptome analysis, . URL https://www.10xgenomics.com/datasets/human-breast-cancer-whole-transcriptome-analysis-1-standard-1-2-0.

[16] Y. Wei, D. Zhang, H. Shi, H. Qian, H. Chen, Q. Zeng, F. Jin, Y. Ye, Z. Ou, M. Guo, B. Guo, and T. Chen. PDK1 promotes breast cancer progression by enhancing the stability and transcriptional activity of HIF-1alpha. 11(4):101041, . ISSN 2352-3042. doi:10.1016/j.gendis.2023.06.013. URL https://www.sciencedirect.com/science/article/pii/S2352304223003112.

[17] D. Zhu, Z. Zhao, G. Cui, S. Chang, L. Hu, Y. X. See, M. G. L. Lim, D. Guo, X. Chen, B. Poudel, P. Robson, Y. Luo, and E. Cheung. Single-cell transcriptome analysis reveals estrogen signaling coordinately augments one-carbon, polyamine, and purine synthesis in breast cancer. 25(8): 2285–2298.e4. ISSN 22111247. doi:10.1016/j.celrep.2018.10.093. URL https://linkinghub.elsevier.com/retrieve/pii/S2211124718317145.

[18] S. Ma, N. Ren, and Q. Huang. rs10514231 leads to breast cancer predisposition by altering ATP6ap1l gene expression. 13(15):3752. ISSN 2072-6694. doi:10.3390/cancers13153752. URL https://www.ncbi.nlm.nih.gov/pmc/articles/PMC8345087/.

[19] A. Shinde, N. Chandak, J. Singh, M. Roy, M. Mane, X. Tang, H. Vasiyani, F. Currim, D. Gohel, S. Shukla, S. Goyani, M. V. Saranga, D. N. Brindley, and R. Singh. TNF-alpha induced NF-KB mediated LYRM7 expression modulates the tumor growth and metastatic ability in breast cancer. 211:158–170. ISSN 0891-5849. doi:10.1016/j.freeradbiomed.2023.12.018. URL https://www.sciencedirect.com/science/article/pii/S0891584923011747.

[20] H. Wu, X. Guo, Y. Jiao, Z. Wu, and Q. Lv. TRIM35 ubiquitination regulates the expression of PKM2 tetramer and dimer and affects the malignant behaviour of breast cancer by regulating the

warburg effect. 61(6):144, . ISSN 1019-6439. doi:10.3892/ijo.2022.5434. URL https://www.ncbi.nlm.nih.gov/pmc/articles/PMC9581112/.

[21] T.-T. Zhao, F. Jin, J.-G. Li, Y.-Y. Xu, H.-T. Dong, Q. Liu, P. Xing, G.-L. Zhu, H. Xu, S.-C. Yin, and Z.-F. Miao. TRIM32 promotes proliferation and confers chemoresistance to breast cancer cells through activation of the NF-KB pathway. 9(8):1349–1356. ISSN 1837-9664. doi:10.7150/jca.22390. URL https://www.ncbi.nlm.nih.gov/pmc/articles/PMC5929078/.

[22] Y.-H. Ko, M. Domingo-Vidal, M. Roche, Z. Lin, D. Whitaker-Menezes, E. Seifert, C. Capparelli, M. Tuluc, R. C. Birbe, P. Tassone, J. M. Curry, A. Navarro-Sabate, A. Manzano, R. Bartrons, J. Caro, and U. Martinez-Outschoorn. TP53-inducible glycolysis and apoptosis regulator (TIGAR) metabolically reprograms carcinoma and stromal cells in breast cancer. 291(51):26291–26303. ISSN 0021-9258. doi:10.1074/jbc.M116.740209. URL https://www.ncbi.nlm.nih.gov/pmc/articles/PMC5159492/.

[23] V. Raghavan and D. B. Manasa. Identification and analysis of disease target network of human MicroRNA and predicting promising leads for ZNF439, a potential target for breast cancer. pages 358–362. ISSN 20103638. doi:10.7763/IJBBB.2012.V2.132. URL http://www.ijbbb.org/show-33-382-1.html.

[24] Y. Salem, N. Yacov, O. Propheta-Meiran, E. Breitbart, and I. Mendel. Newly characterized motile sperm domain-containing protein 2 promotes human breast cancer metastasis. 144(1):125–135. ISSN 1097-0215. doi:10.1002/ijc.31665.

[25] E. J. Suh, M. H. Kabir, U.-B. Kang, J. W. Lee, J. Yu, D.-Y. Noh, and C. Lee. Comparative profiling of plasma proteome from breast cancer patients reveals thrombospondin-1 and BRWD3 as serological biomarkers. 44(1):36–44. ISSN 1226-3613. doi:10.3858/emm.2012.44.1.003. URL https://www.ncbi.nlm.nih.gov/pmc/articles/PMC3277896/.

[26] M. Opławski, A. Srednicka, E. Niewiadomska, D. Boron, P. Januszyk, and B. O. Grabarek. Clinical and molecular evaluation of patients with ovarian cancer in the context of drug resistance to chemotherapy. 12:954008. ISSN 2234-943X. doi:10.3389/fonc.2022.954008. URL https://www.ncbi.nlm.nih.gov/pmc/articles/PMC9389532/.

[27] Z. Xu, X. Li, H. Li, C. Nie, W. Liu, S. Li, Z. Liu, W. Wang, and J. Wang. Suppression of DDX39b sensitizes ovarian cancer cells to DNA-damaging chemotherapeutic agents via destabilizing BRCA1 mRNA. 39(47):7051–7062, . ISSN 1476-5594. doi:10.1038/s41388-020-01482-x. URL https://www.nature.com/articles/s41388-020-01482-x.

[28] L. He, J. Yang, Y. Hao, X. Yang, X. Shi, D. Zhang, D. Zhao, W. Yan, X. Bie, L. Chen, G. Chen, S. Zhao, X. Liu, H. Zheng, and K. Zhang. DDX20: A multifunctional complex protein. 28(20):7198. ISSN 1420-3049. doi:10.3390/molecules28207198. URL https://www.ncbi.nlm.nih.gov/pmc/articles/PMC10608988/.

[29] U.-H. Park, M.-R. Kang, E.-J. Kim, Y.-S. Kwon, W. Hur, S. K. Yoon, B.-J. Song, J. H. Park, J.-T. Hwang, J.-C. Jeong, and S.-J. Um. ASXL2 promotes proliferation of breast cancer cells by linking ERalpha to histone methylation. 35(28):3742–3752. ISSN 1476-5594. doi:10.1038/onc.2015.443. URL https://www.nature.com/articles/onc2015443.

[30] D. Li, Y. Li, X. Wu, Q. Li, J. Yu, J. Gen, and X.-L. Zhang. Knockdown of mgat5 inhibits breast cancer cell growth with activation of CD4+ t cells and macrophages. 180(5):3158–3165, . ISSN 0022-1767, 1550-6606. doi:10.4049/jimmunol.180.5.3158. URL https://journals.aai.org/jimmunol/article/180/5/3158/78739/Knockdown-of-Mgat5-Inhibits-Breast-Cancer-Cell.

[31] P. Wang, Q. Zhang, H. Zhang, J. Shao, H. Zhang, and Z. Wang. Molecular and clinical characterization of ICOS expression in breast cancer through large-scale transcriptome data. 18(12): e0293469, . ISSN 1932-6203. doi:10.1371/journal.pone.0293469. URL https://dx.plos.org/10.1371/journal.pone.0293469.

[32] I. X. Perez-Añorve, C. H. Gonzalez-De la Rosa, E. Soto-Reyes, F. O. Beltran-Anaya, O. Del Moral-Hernandez, M. Salgado-Albarran, O. Angeles-Zaragoza, J. A. Gonzalez-Barrios, D. A. Landero-Huerta, M. Chavez-Saldaña, A. Garcia-Carranca, N. Villegas-Sepulveda, and E. Arechaga-Ocampo. New insights into radioresistance in breast cancer identify a dual function of miR-122 as a tumor suppressor and oncomiR. 13(5):1249–1267. ISSN 1574-7891. doi:10.1002/1878-0261.12483. URL https://www.ncbi.nlm.nih.gov/pmc/articles/PMC6487688/.

[33] C. Tian, S. Zhou, and C. Yi. High NUP43 expression might independently predict poor overall survival in luminal a and in HER2+ breast cancer. 14(15):1431–1442. ISSN 1744-8301. doi:10.2217/fon-2017-0690.

[34] V. Quereda, S. Bayle, F. Vena, S. M. Frydman, A. Monastyrskyi, W. R. Roush, and D. R. Duckett. Therapeutic targeting of CDK12/CDK13 in triple-negative breast cancer. 36(5):545–558.e7. ISSN 15356108. doi:10.1016/j.ccell.2019.09.004. URL https://linkinghub.elsevier.com/retrieve/pii/S1535610819304246.

[35] S. Mohammed Zaidh, K. B. Aher, G. B. Bhavar, N. Irfan, H. N. Ahmed, and Y. Ismail. Genes adaptability and NOL6 protein inhibition studies of fabricated flavan-3-ols lead skeleton intended to treat breast carcinoma. 258:127661. ISSN 0141-8130. doi:10.1016/j.ijbiomac.2023.127661. URL https://www.sciencedirect.com/science/article/pii/S0141813023045592.

[36] B. Arko-Boham, B. A. Owusu, N. A. Aryee, R. M. Blay, E. D. A. Owusu, E. A. Tagoe, A. R. Adams, R. K. Gyasi, N. A. Adu-Aryee, and S. Mahmood. Prospecting for breast cancer blood biomarkers: Death-associated protein kinase 1 (DAPK1) as a potential candidate. 2020:6848703. ISSN 0278-0240. doi:10.1155/2020/6848703. URL https://www.ncbi.nlm.nih.gov/pmc/articles/PMC7267859/.

[37] N. Huang, P. Li, X. Sun, L. Tong, X. Dong, X. Zhang, J. Duan, X. Sheng, and H. Xin. TRIM21 mediates the synergistic effect of olaparib and sorafenib by degrading BRCA1 through ubiquitination in TNBC. 9(1):1–11, . ISSN 2374-4677. doi:10.1038/s41523-023-00588-1. URL https://www.nature.com/articles/s41523-023-00588-1.

[38] W. Li, S. Hu, Z. Han, and X. Jiang. YY1-induced transcriptional activation of FAM111b contributes to the malignancy of breast cancer. 22(4):e417–e425, . ISSN 1526-8209. doi:10.1016/j.clbc.2021.10.008. URL https://www.sciencedirect.com/science/article/pii/S1526820921002986.

[39] X. Chen, H. Peng, Z. Zhang, C. Yang, Y. Liu, Y. Chen, F. Yu, S. Wu, and L. Cao. SPDYC serves as a prognostic biomarker related to lipid metabolism and the immune microenvironment in breast cancer. . ISSN 0257-277X, 1559-0755. doi:10.1007/s12026-024-09505-5. URL https://link.springer.com/10.1007/s12026-024-09505-5.

[40] P. Zhang, Y. Yang, K. Qian, L. Li, C. Zhang, X. Fu, X. Zhang, H. Chen, Q. Liu, S. Cao, and J. Cui. A novel tumor suppressor ZBTB1 regulates tamoxifen resistance and aerobic glycolysis through suppressing HER2 expression in breast cancer. 295(41):14140–14152, . ISSN 00219258. doi:10.1074/jbc.RA119.010759. URL https://linkinghub.elsevier.com/retrieve/pii/S0021925817498097.

[41] X. Wang, Y. Zheng, and Y. Wang. PEAK1 promotes invasion and metastasis and confers drug resistance in breast cancer. 22(3):393–402, . ISSN 1591-9528. doi:10.1007/s10238-021-00761-5. URL https://link.springer.com/10.1007/s10238-021-00761-5.

[42] Y. Peng, X. Liu, X. Liu, X. Cheng, L. Xia, L. Qin, S. Guan, Y. Wang, X. Wu, J. Wu, D. Yan, J. Liu, Y. Zhang, L. Sun, J. Liang, and Y. Shang. RCCD1 promotes breast carcinogenesis through regulating hypoxia-associated mitochondrial homeostasis. 42(50):3684–3697, . ISSN 1476-5594. doi:10.1038/s41388-023-02877-2. URL https://www.nature.com/articles/s41388-023-02877-2.

[43] Y. Niu, Z. Lin, A. Wan, H. Chen, H. Liang, L. Sun, Y. Wang, X. Li, X.-f. Xiong, B. Wei, X. Wu, and G. Wan. RNA n6-methyladenosine demethylase FTO promotes breast tumor progression through inhibiting BNIP3. 18(1):46. ISSN 1476-4598. doi:10.1186/s12943-019-1004-4. URL https://molecular-cancer.biomedcentral.com/articles/10.1186/s12943-019-1004-4.

[44] P. Liang, J. Zhang, Y. Wu, S. Zheng, Z. Xu, S. Yang, J. Wang, S. Ma, L. Xiao, T. Hu, W. Jiang, C. Huang, Q. Xing, M. Kundu, and B. Wang. An ULK1/2-PXN mechanotransduction pathway suppresses breast cancer cell migration. 24(11):e56850. ISSN 1469-221X, 1469-3178. doi:10.15252/embr.202356850. URL https://www.embopress.org/doi/10.15252/embr.202356850.

[45] Y. Lu, Y. Xiao, J. Yang, H. Su, X. Zhang, F. Su, B. Tian, D. Zhao, X. Ling, and T. Zhang. TRIM65 promotes malignant cell behaviors in triple-negative breast cancer by impairing the stability of LATS1 protein. 2022:1–16. ISSN 1942-0994, 1942-0900. doi:10.1155/2022/4374978. URL https://www.hindawi.com/journals/omcl/2022/4374978/.

[46] X. Man, Q. Li, B. Wang, H. Zhang, S. Zhang, and Z. Li. DNMT3a and DNMT3b in breast tumorigenesis and potential therapy. 10:916725. ISSN 2296-634X. doi:10.3389/fcell.2022.916725. URL https://www.frontiersin.org/articles/10.3389/fcell.2022.916725/full.

[47] N. J. Camp, M. Parry, S. Knight, R. Abo, G. Elliott, S. H. Rigas, S. P. Balasubramanian, M. W. R. Reed, H. McBurney, A. Latif, W. G. Newman, L. A. Cannon-Albright, D. G. Evans, and A. Cox. Fine-mapping *CASP8* risk variants in breast cancer. 21(1):176–181. ISSN 1055-9965, 1538-7755. doi:10.1158/1055-9965.EPI-11-0845. URL https://aacrjournals.org/cebp/article/21/1/176/157359/Fine-Mapping-CASP8-Risk-Variants-in-Breast.

[48] J. Xu, S. M. Su, X. Zhang, U. I. Chan, R. Adhav, X. Shu, J. Liu, J. Li, L. Mo, Y. Wang, T. An, J. H. Lei, K. Miao, C.-X. Deng, and X. Xu. ATP11b inhibits breast cancer metastasis in a mouse model by suppressing externalization of nonapoptotic phosphatidylserine. 132(5):e149473, . ISSN 1558-8238. doi:10.1172/JCI149473. URL https://www.jci.org/articles/view/149473.

[49] S. Kim, K. Kim, J. Ryu, T. Ryu, J. H. Lim, J. Oh, J. Min, C. Jung, R. Hamamoto, M. Son, D. Kim, and H. Cho. The novel prognostic marker, EHMT2, is involved in cell proliferation via HSPD1 regulation in breast cancer. ISSN 1019-6439, 1791-2423. doi:10.3892/ijo.2018.4608. URL http://www.spandidos-publications.com/10.3892/ijo.2018.4608.

[50] A. K. Pullikuth, E. D. Routh, K. D. Zimmerman, J. Chifman, J. W. Chou, M. H. Soike, G. Jin, J. Su, Q. Song, M. A. Black, C. Print, D. Bedognetti, M. Howard-McNatt, S. S. O'Neill, A. Thomas, C. D. Langefeld, A. B. Sigalov, Y. Lu, and L. D. Miller. Bulk and single-cell profiling of breast tumors identifies TREM-1 as a dominant immune suppressive marker associated with poor outcomes. 11:734959. ISSN 2234-943X. doi:10.3389/fonc.2021.734959. URL https://www.frontiersin.org/articles/10.3389/fonc.2021.734959/full.

[51] L. Pei, Y. Li, H. Gu, S. Wang, W. Wu, S. Fan, X. Shi, and X. Si. Identification of SMC2 and SMC4 as prognostic markers in breast cancer through bioinformatics analysis. ISSN 1699-3055. doi:10.1007/s12094-024-03521-5. URL https://link.springer.com/10.1007/s12094-024-03521-5.

[52] M. Viera, G. W. C. Yip, H.-M. Shen, G. H. Baeg, and B. H. Bay. Targeting CD82/KAI1 for precision therapeutics in surmounting metastatic potential in breast cancer. 13(17):4486. ISSN 2072-6694. doi:10.3390/cancers13174486. URL https://www.mdpi.com/2072-6694/13/17/4486.

[53] D. Samanta, T. Y.-T. Huang, R. Shah, Y. Yang, F. Pan, and G. L. Semenza. BIRC2 expression impairs anti-cancer immunity and immunotherapy efficacy. 32(8):108073. ISSN 22111247. doi:10.1016/j.celrep.2020.108073. URL https://linkinghub.elsevier.com/retrieve/pii/S2211124720310585.

[54] E. Hervouet, A. Claude-Taupin, T. Gauthier, V. Perez, A. Fraichard, P. Adami, G. Despouy, F. Monnien, M.-P. Algros, M. Jouvenot, R. Delage-Mourroux, and M. Boyer-Guittaut. The autophagy GABARAPL1 gene is epigenetically regulated in breast cancer models. 15(1):729. ISSN 1471-2407. doi:10.1186/s12885-015-1761-4. URL http://bmccancer.biomedcentral.com/articles/10.1186/s12885-015-1761-4.

[55] M. Siouda, A. D. Dujardin, L. Barbollat-Boutrand, M. A. Mendoza-Parra, B. Gibert, M. Ouzounova, J. Bouaoud, L. Tonon, M. Robert, J.-P. Foy, V. Lavergne, S. N. Manie, A. Viari, A. Puisieux, G. Ichim, H. Gronemeyer, P. Saintigny, and P. Mulligan. CDYL2 epigenetically regulates MIR124 to control NF-KB/STAT3-dependent breast cancer cell plasticity. 23(6):101141. ISSN 25890042. doi:10.1016/j.isci.2020.101141. URL https://linkinghub.elsevier.com/retrieve/pii/S2589004220303266.

[56] M.-H. Kang, K. J. Jeong, W. Y. Kim, H. J. Lee, G. Gong, N. Suh, B. Győrffy, S. Kim, S.-Y. Jeong, G. B. Mills, and Y.-Y. Park. Musashi RNA-binding protein 2 regulates estrogen receptor 1 function in breast cancer. 36(12):1745–1752. ISSN 0950-9232, 1476-5594. doi:10.1038/onc.2016.327. URL https://www.nature.com/articles/onc2016327.

[57] W.-F. Hu, K. L. Krieger, D. Lagundžin, X. Li, R. S. Cheung, T. Taniguchi, K. R. Johnson, T. Bessho, A. N. A. Monteiro, and N. T. Woods. CTDP1 regulates breast cancer survival and DNA repair through BRCT-specific interactions with FANCI. 5(1):105. ISSN 2058-7716. doi:10.1038/s41420-019-0185-3. URL https://www.nature.com/articles/s41420-019-0185-3.

[58] J. Zhang, Y. Li, J.-G. Wang, J.-Y. Feng, G.-D. Huang, and C.-G. Luo. Dihydroartemisinin affects STAT3/DDA1 signaling pathway and reverses breast cancer resistance to cisplatin. 51(2):445–459, . ISSN 0192-415X, 1793-6853. doi:10.1142/S0192415X23500234. URL https://www.worldscientific.com/doi/10.1142/S0192415X23500234.

[59] Y. Peng, H. Li, Y. Fu, S. Guo, C. Qu, Y. Zhang, B. Zong, and S. Liu. JAM2 predicts a good prognosis and inhibits invasion and migration by suppressing EMT pathway in breast cancer. 103: 108430, . ISSN 15675769. doi:10.1016/j.intimp.2021.108430. URL https://linkinghub.elsevier.com/retrieve/pii/S1567576921010663.

[60] S. Zhang, X. Liu, W. Chen, K. Zhang, Q. Wu, and Y. Wei. Targeting TAF1 with BAY-299 induces antitumor immunity in triple-negative breast cancer. 665:55–63, . ISSN 0006291X. doi:10.1016/j.bbrc.2023.04.100. URL https://linkinghub.elsevier.com/retrieve/pii/S0006291X23005314.

[61] C. N. Johnstone, A. D. Pattison, P. F. Harrison, D. R. Powell, P. Lock, M. Ernst, R. L. Anderson, and T. H. Beilharz. FGF13 promotes metastasis of triple-negative breast cancer. 147(1):230–243. ISSN 0020-7136, 1097-0215. doi:10.1002/ijc.32874. URL https://onlinelibrary.wiley.com/doi/10.1002/ijc.32874.

[62] Z. Chen, N. Cui, J.-s. Zhao, J.-f. Wu, F. Ma, C. Li, and X.-y. Liu. Expressions of ZNF436, beta-catenin, EGFR, and CMTM5 in breast cancer and their clinical significances. 65(1), . ISSN 2038-8306, 1121-760X. doi:10.4081/ejh.2021.3173. URL https://www.ejh.it/index.php/ejh/article/view/3173.

[63] M. Ngubo, F. Moradi, C. Y. Ito, and W. L. Stanford. Tissue-specific tumour suppressor and oncogenic activities of the polycomb-like protein MTF2. 14(10):1879. ISSN 2073-4425. doi:10.3390/genes14101879. URL https://www.mdpi.com/2073-4425/14/10/1879.

[64] L. Jin, C. Luo, X. Wu, M. Li, S. Wu, and Y. Feng. LncRNA-HAGLR motivates triple negative breast cancer progression by regulation of WNT2 via sponging miR-335-3p. 13(15):19306–19316, . ISSN 1945-4589. doi:10.18632/aging.203272. URL https://www.aging-us.com/lookup/doi/10.18632/aging.203272.

[65] H.-J. Han, J. Russo, Y. Kohwi, and T. Kohwi-Shigematsu. SATB1 reprogrammes gene expression to promote breast tumour growth and metastasis. 452(7184):187–193. ISSN 0028-0836, 1476-4687. doi:10.1038/nature06781. URL https://www.nature.com/articles/nature06781.

[66] N. Erin, A. Podnos, G. Tanriover, O. Duymus, E. Cote, I. Khatri, and R. M. Gorczynski. Bidirectional effect of CD200 on breast cancer development and metastasis, with ultimate outcome determined by tumor aggressiveness and a cancer-induced inflammatory response. 34(29):3860–3870. ISSN 0950-9232, 1476-5594. doi:10.1038/onc.2014.317. URL https://www.nature.com/articles/onc2014317.

[67] P. Tan, Y. Ye, L. He, J. Xie, J. Jing, G. Ma, H. Pan, L. Han, W. Han, and Y. Zhou. TRIM59 promotes breast cancer motility by suppressing p62-selective autophagic degradation of PDCD10. 16 (11):e3000051. ISSN 1545-7885. doi:10.1371/journal.pbio.3000051. URL https://dx.plos.org/10.1371/journal.pbio.3000051.

[68] M. Mondal, D. Conole, J. Nautiyal, and E. W. Tate. UCHL1 as a novel target in breast cancer: emerging insights from cell and chemical biology. 126(1):24–33. ISSN 0007-0920, 1532-1827. doi:10.1038/s41416-021-01516-5. URL https://www.nature.com/articles/s41416-021-01516-5.

[69] D. Chen, Y. Sun, Y. Wei, P. Zhang, A. H. Rezaeian, J. Teruya-Feldstein, S. Gupta, H. Liang, H.-K. Lin, M.-C. Hung, and L. Ma. LIFR is a breast cancer metastasis suppressor upstream of the

hippo-YAP pathway and a prognostic marker. 18(10):1511–1517, . ISSN 1078-8956, 1546-170X. doi:10.1038/nm.2940. URL https://www.nature.com/articles/nm.2940.

[70] I. A. Ivanova, J. F. Vermeulen, C. Ercan, J. M. Houthuijzen, F. A. Saig, E. J. Vlug, E. Van Der Wall, P. J. Van Diest, M. Vooijs, and P. W. B. Derksen. FER kinase promotes breast cancer metastasis by regulating alpha6- and beta1-integrin-dependent cell adhesion and anoikis resistance. 32(50):5582–5592. ISSN 0950-9232, 1476-5594. doi:10.1038/onc.2013.277. URL https://www.nature.com/articles/onc2013277.

[71] W. Yang, J. Li, M. Zhang, H. Yu, Y. Zhuang, L. Zhao, L. Ren, J. Gong, H. Bi, L. Zeng, Y. Xue, J. Yang, Y. Zhao, S. Wang, S. Gao, Z. Fu, D. Li, J. Zhang, T. Wang, M. Shan, B. Tang, and X. Li. Elevated expression of the rhythm gene NFIL3 promotes the progression of TNBC by activating NF-KB signaling through suppression of NFKBIA transcription. 41(1):67, . ISSN 1756-9966. doi:10.1186/s13046-022-02260-1. URL https://jeccr.biomedcentral.com/articles/10.1186/s13046-022-02260-1.

[72] D. B. Shropshire, F. M. Acosta, K. Fang, J. Benavides, L.-Z. Sun, V. X. Jin, and J. X. Jiang. Association of adenosine signaling gene signature with estrogen receptor-positive breast and prostate cancer bone metastasis. 9:965429. ISSN 2296-858X. doi:10.3389/fmed.2022.965429. URL https://www.frontiersin.org/articles/10.3389/fmed.2022.965429/full.

[73] A. M. Marrufo, S. O. Mathew, P. Chaudhary, J. D. Malaer, J. K. Vishwanatha, and P. A. Mathew. Blocking LLT1 (CLEC2d, OCIL)-NKRP1a (CD161) interaction enhances natural killer cell-mediated lysis of triple-negative breast cancer cells. 8(6):1050–1063. ISSN 2156-6976. URL https://www.ncbi.nlm.nih.gov/pmc/articles/PMC6048397/.

[74] Y. Tai, A. Chow, S. Han, C. Coker, W. Ma, Y. Gu, V. Estrada Navarro, M. Kandpal, H. Hibshoosh, K. Kalinsky, K. Manova-Todorova, A. Safonov, E. M. Walsh, M. Robson, L. Norton, R. Baer, T. Merghoub, A. K. Biswas, and S. Acharyya. FLT1 activation in cancer cells promotes PARP-inhibitor resistance in breast cancer. 16(8):1957–1980. ISSN 1757-4684. doi:10.1038/s44321-024-00094-2. URL https://www.embopress.org/doi/full/10.1038/s44321-024-00094-2.

[75] Y.-J. Lee, S.-R. Ho, J. D. Graves, Y. Xiao, S. Huang, and W.-C. Lin. CGRRF1, a growth suppressor, regulates EGFR ubiquitination in breast cancer. 21(1):134. ISSN 1465-542X. doi:10.1186/s13058-019-1212-2. URL https://breast-cancer-research.biomedcentral.com/articles/10.1186/s13058-019-1212-2.

[76] R. C. Ekyalongo, T. Mukohara, Y. Funakoshi, H. Tomioka, Y. Kataoka, Y. Shimono, N. Chayahara, M. Toyoda, N. Kiyota, and H. Minami. TYRO3 as a potential therapeutic target in breast cancer. 34(7):3337–3345. ISSN 1791-7530.

[77] Y. Cheng, L. Lin, X. Li, A. Lu, C. Hou, Q. Wu, X. Hu, Z. Zhou, Z. Chen, and F. Tang. ADAM10 is involved in the oncogenic process and chemo-resistance of triple-negative breast cancer via regulating notch1 signaling pathway, CD44 and PrPc. 21(1):32. ISSN 1475-2867. doi:10.1186/s12935-020-01727-5. URL https://cancerci.biomedcentral.com/articles/10.1186/s12935-020-01727-5.

[78] Y. Wei, H. Huang, Z. Qiu, H. Li, J. Tan, G. Ren, and X. Wang. NLRP1 overexpression is correlated with the tumorigenesis and proliferation of human breast tumor. 2017:1–9, . ISSN 2314-6133,

2314-6141. doi:10.1155/2017/4938473. URL
https://www.hindawi.com/journals/bmri/2017/4938473/.

[79] Z. Huang, J. Yang, W. Qiu, J. Huang, Z. Chen, Y. Han, and C. Ye. HAUS5 is a potential prognostic biomarker with functional significance in breast cancer. 12:829777, . ISSN 2234-943X. doi:10.3389/fonc.2022.829777. URL https://www.frontiersin.org/articles/10.3389/fonc.2022.829777/full.

[80] D. Tong, J. Zhang, X. Wang, Q. Li, L. Y. Liu, J. Yang, B. Guo, L. Ni, L. Zhao, and C. Huang. MeCP2 facilitates breast cancer growth via promoting ubiquitination-mediated p53 degradation by inhibiting RPL5/RPL11 transcription. 9(5):56. ISSN 2157-9024. doi:10.1038/s41389-020-0239-7. URL https://www.nature.com/articles/s41389-020-0239-7.

[81] M. Spears, G. J. Rabiasz, D. Scott, E. Ntougkos, S. Fegan, E. P. Miller, J. F. Smyth, and G. C. Sellar. The function of tumor suppressor genes in ovarian cancer: the role of LSAMP. 66 (8_Supplement)(587). ISSN 1538-7445.

[82] Y. Shi, S. Gao, Y. Zheng, M. Yao, and F. Ruan. LncRNA CASC15 functions as an unfavorable predictor of ovarian cancer prognosis and inhibits tumor progression through regulation of miR-221/ARID1a axis. 12:8725–8736. ISSN 1178-6930. doi:10.2147/OTT.S219900. URL https://www.ncbi.nlm.nih.gov/pmc/articles/PMC6815787/.

[83] A.-r. Liu, Y.-n. Liu, S.-x. Shen, L.-r. Yan, Z. Lv, H.-x. Ding, A. Wang, Y. Yuan, and Q. Xu. Comprehensive analysis and validation of solute carrier family 25 (SLC25) and its correlation with immune infiltration in pan-cancer. 2022:1–23. ISSN 2314-6141, 2314-6133. doi:10.1155/2022/4009354. URL https://www.hindawi.com/journals/bmri/2022/4009354/.

[84] J. Wang, D. C. Dean, F. J. Hornicek, H. Shi, and Z. Duan. Cyclin-dependent kinase 9 (CDK9) is a novel prognostic marker and therapeutic target in ovarian cancer. 33(5):5990–6000, . ISSN 0892-6638, 1530-6860. doi:10.1096/fj.201801789RR. URL https://onlinelibrary.wiley.com/doi/10.1096/fj.201801789RR.

[85] T. Gruosso, C. Garnier, S. Abelanet, Y. Kieffer, V. Lemesre, D. Bellanger, I. Bieche, E. Marangoni, X. Sastre-Garau, V. Mieulet, and F. Mechta-Grigoriou. MAP3k8/TPL-2/COT is a potential predictive marker for MEK inhibitor treatment in high-grade serous ovarian carcinomas. 6(1):8583. ISSN 2041-1723. doi:10.1038/ncomms9583. URL https://www.nature.com/articles/ncomms9583.

[86] S. Wang, Y. Xia, P. Huang, C. Xu, Y. Qian, T. Fang, and Q. Gao. Suppression of GCH1 sensitizes ovarian cancer and breast cancer to PARP inhibitor. 2023:1–16, . ISSN 1687-8469, 1687-8450. doi:10.1155/2023/1453739. URL https://www.hindawi.com/journals/jo/2023/1453739/.

[87] Y. Xie, H. Chen, P. Shen, Q. Shen, and Y. Luo. PCYT2-mediated regulation of phospholipid metabolism enhances metastasis in epithelial ovarian cancer via the AKT/mTOR and HIPPO signaling pathways. 38(2):1351–1364. ISSN 0393-974X. doi:10.23812/j.biol.regul.homeost.agents.20243802.108. URL https://www.biolifesas.org/EN/10.23812/j.biol.regul.homeost.agents.20243802.108.
